# Single-Cell Transcriptome Analysis Reveals Six Subpopulations Reflecting Distinct Cellular Fates in Senescent Mouse Embryonic Fibroblasts

**DOI:** 10.3389/fgene.2020.00867

**Published:** 2020-08-11

**Authors:** Wei Chen, Xuefei Wang, Gang Wei, Yin Huang, Yufang Shi, Dan Li, Shengnu Qiu, Bin Zhou, Junhong Cao, Meng Chen, Pengfei Qin, Wenfei Jin, Ting Ni

**Affiliations:** ^1^State Key Laboratory of Genetic Engineering, Collaborative Innovation Center of Genetics and Development, School of Life Sciences, Human Phenome Institute, Fudan University, Shanghai, China; ^2^Department of Biology, Southern University of Science and Technology, Shenzhen, China; ^3^Shanghai Institute of Nutrition and Health, Shanghai Institutes for Biological Sciences, Chinese Academy of Sciences, University of Chinese Academy of Sciences, Shanghai, China; ^4^The First Affiliated Hospital of Soochow University and State Key Laboratory of Radiation Medicine and Protection, Institutes for Translational Medicine, Soochow University, Suzhou, China; ^5^Field Application Department, Fluidigm (Shanghai) Instrument Technology Co., Ltd., Shanghai, China; ^6^Division of Biosciences, Faculty of Life Sciences, University College London, London, United Kingdom; ^7^Eye Institute, Eye & ENT Hospital, Shanghai Medical College, Fudan University, Shanghai, China

**Keywords:** cellular senescence, single-cell RNA sequencing, mouse embryonic fibroblasts, transcriptomic heterogeneity, senescence-associated secretory phenotype, Hoxd8

## Abstract

Replicative senescence is a hallmark of aging, which also contributes to individual aging. Mouse embryonic fibroblasts (MEFs) provide a convenient replicative senescence model. However, the heterogeneity of single MEFs during cellular senescence has remained unclear. Here, we conducted single-cell RNA sequencing on senescent MEFs. Principal component analysis showed obvious heterogeneity among these MEFs such that they could be divided into six subpopulations. Three types of gene expression analysis revealed distinct expression features of these six subpopulations. Trajectory analysis revealed three distinct lineages during MEF senescence. In the main lineage, some senescence-associated secretory phenotypes were upregulated in a subset of cells from senescent clusters, which could not be distinguished in a previous bulk study. In the other two lineages, a possibility of escape from cell cycle arrest and coupling between translation-related genes and ATP synthesis-related genes were also discovered. Additionally, we found co-expression of transcription factor HOXD8 coding gene and its potential target genes in the main lineage. Overexpression of *Hoxd8* led to senescence-associated phenotypes, suggesting HOXD8 is a new regulator of MEF senescence. Together, our single-cell sequencing on senescent MEFs largely expanded the knowledge of a basic cell model for aging research.

## Introduction

Cellular senescence was first discovered by Hayflick and Moorhead (1961) in normal human fibroblasts during a study in which they observed a finite replicative life span. The mechanism of this phenomenon was subsequently explained as gradual shortening and loss of telomeres in the absence of telomerase (Olovnikov, 1996). Loss of telomeres triggers the DNA damage response. Proteins involved in DNA damage response (e.g., ATM, Chk1, and Chk2) activates cell cycle proteins p53 and p21, which then inhibit a subset of cyclin-dependent kinases (CDKs) and lead to cell cycle arrest (Leonardo et al., 1994; Herbig et al., 2004). In addition to replicative exhaustion, other factors such as DNA damage, oncogene hyperactivation, oxidative stress, and chromatin perturbation can also cause cellular senescence. These factors are considered “stress” and can induce premature cellular senescence. DNA damage can be caused by external agents, such as H_2_O_2_, X-ray or ultraviolet irradiation, and many chemotherapeutic drugs. The pathways activated in this context are similar to those activated during telomere erosion. In oncogene-induced cellular senescence, overexpression (OE) of certain oncogenes causes a DNA damage response or upregulation of alternative reading frame (ARF) (belongs to CDKN2A locus), which activates the p53-p21 pathway or p16 (Campisi and Fagagna, 2007). In contrast to replicative senescence, oncogene-induced senescence (OIS) is telomere-independent and much more acute (Gorgoulis and Halazonetis, 2010). In mouse embryonic fibroblasts (MEFs), telomeres are much longer (40–60 kb) than those in human cells (5–15 kb). Cellular senescence that occurs in MEFs is a response to oxidative stress, instead of telomere erosion (Sherr and Dipinho, 2000; Wright and Shay, 2000). MEFs cultured in standard conditions (20% oxygen) show substantial DNA damage, whereas physical oxygen concentration (3% oxygen) does not trigger cellular senescence in MEFs (Parrinello et al., 2003). Exposure of MEFs to oxidative stress activates the p19/ARF-p53 pathway, which leads to cellular senescence (Harvey et al., 1993; Kamijo et al., 1997). In summary, replicative senescence is intrinsic and acts as an intracellular mitotic clock in the cell, whereas stress-induced senescence is extrinsic.

Based on the notion that tumorigenesis is the result of escape of a cell’s regular senescence fate of a cell, replicative senescence can be regarded as an important cancer prevention mechanism (Elkon et al., 2009). A recent study demonstrated that the *in vivo* induction of senescence in cancer cells attracts natural killer cells to clear the cancer cells; thus, this senescence is beneficial to immunotherapy (Ruscetti et al., 2018). Senescence-associated secretory phenotype (SASP) components released by senescent cancer cells mediate such clearance by immune cells. Replicative senescence also contributes to individual aging (López-Otín et al., 2013). Accumulation of senescent cells in aged tissues/organs leads to a considerable release of SASP components into the local environment, which promotes senescence of nearby cells in a paracrine fashion and ultimately results in tissue/organ dysfunction (Dimri et al., 1995; Muñoz-Espín and Serrano, 2014). Thus, clearance of senescent cells in the mouse model benefits tissue function and increases health span (Baker et al., 2016).

Studies of cellular senescence have been performed using normal human fibroblasts (Hayflick and Moorhead, 1961), human diploid keratinocytes (Rheinwald and Green, 1975), human vascular smooth muscle cells (Bierman, 1978), human lens cells (Tassin et al., 1979), and human peripheral lymphocyte (Tice et al., 1979), as well as a variety of other cells. MEFs have a relatively short cultivation time *in vitro* (typically 15–30 population doublings) and thus serve as a time-saving model to study cellular senescence (Sherr and Dipinho, 2000). Previous studies illustrated that *in vitro* cultivated senescent MEFs manifested upregulation of *Cdkn1a* (encoding p21), *Cdkn2a* (encoding p16), *Lmnb1*, *Mki67*, and other conventional senescence markers (Sherr and Dipinho, 2000; Parrinello et al., 2003; Itahana et al., 2004). This phenotype was similar to that of senescence in human fibroblasts (Kim et al., 2013). Significant enhancement of senescence-associated β-galactosidase (SA-β-Gal) staining was also detected in senescent MEFs, mimicking the same traditional senescence marker in human cells (Dimri et al., 1995). Furthermore, primary MEF cells have been used for lentivirus-mediated gene knockdown or OE experiments, facilitating the mechanistic investigation of candidate genes’ impact on senescence (Xu et al., 2019). Importantly, findings discovered in cultured MEFs can also be extended *in vivo*. For example, the transforming growth factor (TGF)-β/miR-29 pathway that originally revealed in MEFs was further proven to contribute to cardiac aging in a mouse model (Lyu et al., 2018). Therefore, MEFs can be used as an alternative model for investigation of cellular senescence.

In the past decade, various single-cell RNA sequencing (scRNA-seq) methods were developed and revealed the heterogeneity of seemingly identical cells, emphasizing minute differences between single cells in immune cells, embryonic cells, neurons, and others (Tang et al., 2010; Xue et al., 2013; Deng et al., 2014). Some studies applied scRNA-seq to aging and/or senescence. One study reported that upon activation, CD4^+^ T cells from old (21 months) B6 or CAST mice displayed higher cell-to-cell transcriptomic variability of the core activation program compared with cells from their young (3 months) counterparts; this increasing transcriptomic variability was independent of expression level alteration (Martinez-Jimenez et al., 2017). Another study illustrated that hematopoietic stem cells from old mice have a higher rate of self-renewal and a lower rate of differentiation, compared with those cells from younger mice, based on single-cell transcriptome analysis (Kowalczyk et al., 2015). By combining Hi-C, single-cell and bulk RNA-seq, as well as imaging in proliferating and senescent human umbilical vein endothelial cells (HUVECs), Zirkel et al. (2018) found that high mobility group box 2 (HMGB2) loss during early senescence led to genomic reorganization and senescence-induced CCCTC-Binding Factor (CTCF) clustering. Despite such progress regarding aging-related single-cell RNA-seq analyses, some basic questions remain elusive in replicative senescence models such as MEFs. For example: (1) What is the degree of heterogeneity in MEF senescence? (2) Does SASP contribute to MEF senescence? (3) Could scRNA-seq aid in the discovery of novel senescence regulators in MEFs?

To address these questions, we performed single-cell full-length RNA-seq in atmospheric (20%) oxygen-cultivated MEFs at a passage where half of the cells were positive in SA-β-Gal staining. We observed considerable variation in gene expression levels, although these cells were obtained from the same passage. Six clusters and three distinct lineages were detected, which exhibited specific molecular features during senescence. In contrast to the finding of a previous bulk study, we discovered that some SASP components were upregulated in a few single MEFs from senescent clusters. Additionally, OE of *Hoxd8* led to several senescence-associated phenotypes. This study provides a new perspective for understanding the basic features of an important senescence model.

## Materials and Methods

### Cell Isolation and *in vitro* Cultivation

Mouse embryos were taken from 12.5∼14.5 days of pregnant C57BL/6, and primary MEF cells were isolated following a previously described protocol (Todaro and Green, 1963). NIH3T3 cells were provided by the American Type Culture Collection (ATCC, Manassas, VA, United States). Cells were cultivated *in vitro* in Dulbecco’s modified Eagle’s medium (DMEM) medium (Gibco) with 10% fetal bovine serum (FBS; Gibco) in 25-cm^2^ flasks, which were placed in an incubator with 5% CO_2_ and 37•C. Once the confluence reached 70% in the flask, cells were resuspended by 0.25% trypsin-EDTA (Gibco) and evenly divided into two new flasks. Population doubling (PD) was added by 1 each time MEF cells were subcultured.

### Single-Cell RNA Sequencing

PD9 MEF cells were collected. Cell counting was performed in Cellometer Mimi (Nexcelom Bioscience). Single cells were added into three 17–25-μm Single-Cell mRNA Seq IFC (Fluidigm C1). After loading into the chip, cells were imaged on the microscope to filter out wells with no cell, cell doublet, or cell debris. Full-length cDNA libraries were auto-constructed in Fluidigm C1 system using SMART-seq v4 kits. Quality control was carried out on each single-cell cDNA library using Qubit 3.0 and Aligent Bioanalyzer 2100 to exclude libraries with abnormal molecular features. Sequencing libraries were constructed using Nextera XT DNA library kit, and another round of quality control was performed. RNA-seq libraries were then pooled and sequenced by Illumina Hiseq 4000 with an average depth of 3 million reads for each single cell.

### Paracrine Experiments

For total SASP experiments, primary MEF cells were *in vitro* cultivated to PD11. PD11 MEF cells were cultivated with DMEM medium (10% FBS) for 2 days, and senescence-conditioned medium (SCM) was collected. Then, SCM was centrifuged at 3,000 rpm for 5 min and filtered through a 0.45-μm syringe filter. After that, newly thawed primary MEF cells were evenly distributed into two flasks and cultured with normal medium (NM) and SCM simultaneously. For interleukin (IL)6 experiments, we bought recombinant mouse IL6 from R&D Systems (Bio-Techne). Newly thawed primary MEF cells were evenly distributed into three flasks and cultured with DMEM (10% FBS), DMEM (10% FBS and 5 ng/ml IL6), and DMEM (10% FBS and 50 ng/ml IL6) simultaneously. SA-β-Gal staining, cell cycle analysis, RNA extraction, quantitative reverse transcription PCR (qRT-PCR), and RNA-seq were conducted on these three different treated MEFs.

### Senescence-Associated β-Galactosidase Staining

Proper amount of cells were seeded into multiple wells in a 12-well plate and cultivated for 24 h. Then, SA-β-Gal staining was performed for MEFs with different conditions using Senescence Cells Histochemical Staining Kit (Sigma) following its manual. Photos were taken under an inverted microscope.

### Cell Cycle Analysis

One fourth of the cells in a flask with 70% confluence was lysed and washed twice with 1 × phosphate buffered saline (PBS). Then, cells were permeabilized with 50 μg/ml Triton-X100 (Sigma) and stained with 0.15% propidium iodide (Thermal Fisher) in the dark for 10 min. Cells were then loaded into Calibur flow cytometer (Becton Dickinson) to measure the fluorescence. Three replicates for each sample were performed. Results were analyzed by ModFit software.

### Ethynyl-2′-Deoxyuridine Incorporation Assay

The proper amount of cells was seeded into multiple wells in a 24-well plate. Cells were cultivated until the confluence reached 50%. Then, culture medium was aspirated, and cell was incubated at 10 μM 5-ethynyl-2′-deoxyuridine (EdU) for 2 h. Cell fixation, permeation, and staining with fluorescence dyes were implemented following keyFluor488 Click-iT Edu image kit manual (KeyGEN). Fluorescence was examined under IX73 fluorescence microscope (Olympus) with 495 nm (kFluor488) and 350 nm (Hochest 33342) excitation filter. Photos shot on the same field were merged.

### Cell Proliferation Rate Assay

A total of 2,000 cells were seeded into each well of a 96-well plate. For each sample, five replicates were applied (five wells per sample). Cells were then cultivated at an incubator for 24, 48, 72, and 96 h. After cultivation, cell medium was aspirated, and cells were incubated at 10% Cell Counting Kit-8 solution (Dojindo) at 37•C for 3 h. Absorbance at 450 and 630 nm were detected at Bio-Rad 680 microplate reader.

### Quantitative Reverse Transcription PCR and Bulk Population RNA Sequencing

Total RNA was extracted by TRIzol reagent (Life Technology) following its manual. For qRT-PCR, genomic DNA depletion and reverse transcription were performed using HiScript II Q RT SuperMix for qPCR (Vazyme) following its manual. qPCR mix was composed of 1 μl 1:5 diluted cDNA product, 0.4 μl 10 μM F/R primer, 10 μl 2 × ChamQ Universal SYBR qPCR Master Mix (Vazyme), and 8.2 μl nuclease-free water. Three replicates were applied for each sample. Then, qPCR was performed in CFX Connect Real-Time PCR Detection System (Bio-Rad) following Vazyme’s program: one cycle of 95•C 30 s, followed by 40 cycles of 95•C 10 s and 60•C 30 s, finally one cycle of 95•C 15 s, 60•C 60 s, and 95•C 15 s. For bulk population RNA-seq, 1 μg total RNA was used as starting material. Libraries were constructed using KAPA RNA HyperPrep Kit (Roche) following its manual. Paired-End 150-bp (PE-150) sequencing was performed at Illumina Hiseq 2500 platform with an average depth of 30 million reads for each sample.

### Hoxd8 Overexpression

Total RNA from mouse NIH3T3 cell was reversely transcribed by oligo dT(20) primer. The resulting cDNA was amplified by gene-specific PCR primer to obtain full-length cDNA of *Hoxd8* and ligated into pCDH vector to make the OE vector. The vector was sequenced to ensure 100% accuracy of inserted *Hoxd8* cDNA. After vector construction, lentivirus was assembled in 293T cells. Culture medium were centrifuged at 3,000 rpm for 5 min and filtered through a 0.45-μm syringe filter to obtain lentivirus. Then, NIH3T3 cells were infected with lentivirus containing *Hoxd8*. Uninfected cells were filtered out by adding Puro (10 mg/ml, Sangon Biotech) into the culture medium. The OE of *Hoxd8* was confirmed by qRT-PCR, and multiple senescence-associated phenotypes were then examined.

### Bulk-Population RNA Sequencing Data Analysis

Adapter trimming was performed by cutadapt for RNA sequencing reads in FASTQ-format files. Trimmed sequencing reads were aligned to annotated reference genome (NCBI RefSeq annotations for genomes mm10) by STAR following the default parameters (e.g., “runMode” as genome Generate mode, “allows varying length with parameter sjdbOverhang” as 149, “outFilterMultimapNmax” as 10; Dobin et al., 2013). The reads per kilobase million (RPKM) of genes were quantified by FeatureCounts (Liao et al., 2014), which took paired-end reads into consideration, and then normalized by gene length. To compare the difference between bulk population RNA-seq and scRNA-seq, gene expression level of scRNA-seq was averaged for each cluster of cells.

### Dimension Reduction and Clustering Analysis

RNA sequencing reads were adapter trimmed by cutadapt. Trimmed sequencing reads were further aligned to reference genome using STAR with parameters similar with those as described above. RPKM of genes was quantified by FeatureCounts and normalized by gene length. Cells with extremely low abundance of valid reads were removed. Dimension reduction, namely, principal component analysis (PCA) and T-distributed stochastic neighbor embedding (tSNE), was performed using Seurat v3 (Macosko et al., 2015). The highly variable genes (HVGs) were selected based on normalized dispersion following Macosko et al. (2015). We used around 2,500, 5,000, 8,000 HVGs, respectively, to perform dimension reduction and clustering analysis in order to get reliable results. Because the clustering results were similar, we chose to use around 2,500 HVGs. Outliers were further determined and filtered by PCA projection. About top 50 principal components (PCs) were chosen for tSNE projection (Maaten and Hinton, 2012). Clustering of single cells was performed by KNN graph construction and Louvain algorithm. Considering the sample size in our case, parameter k in clustering algorithm was set as 10. Parameter of resolution was set from 0.8 to 2 to screen all possible clusters. Differentially expressed gene (DEG) identification of each cluster was performed using all genes following the pipeline of a previous paper (McDavid et al., 2013). Gene Ontology (GO) analysis was performed in Metascape (Zhou et al., 2019). Raw clusters with few DEGs were merged to avoid excessive classification. At last perplexity in tSNE was set as 10.

### Expressed Gene Selection of Single-Cell RNA Sequencing and Bulk Population RNA Sequencing Data on Mouse Embryonic Fibroblasts

The bulk population RNA-seq data of transcriptomes of *in vitro* cultivated MEFs from PD6 to PD11 was obtained from our previous paper (Chen et al., 2018). The expressed genes in scRNA-seq were filtered by criteria of log (exp) > 0.01, which is the 10% quantile of expression level. For bulk RNA data, we only considered genes of > 1 fragment per kilobase million (FPKM) in all the *in vitro* cultivation passages from PD6 to PD11.

### Gene Set Enrichment Analysis

Gene set enrichment analysis (GSEA) measures the enrichment of *a priori* defined set of genes. We implemented a GSEA approach for single cells, following steps as below: (1) Genes were ranked according to their expression level for each cell. (2) Recovery curve was created by walking down *a priori* defined gene list, and steps were increased when we encounter a gene in the gene set. The area under the curve (AUC) was computed as the indicator of enrichment for a certain gene set. And only AUC of top 3,000 ranked genes was considered. AUC values were normalized across cells.

### Cell Cycle-Related Genes, Immune and Senescence-Related Genes, and Translation-Related Genes

The list of cell cycle-related genes in mouse was obtained from the previous paper “Highly Parallel Genome Wide Expression Profiling of Individual Cells Using Nanoliter Droplets” (Macosko et al., 2015). Immune and senescence-related genes were chosen from immune and senescence-related GO terms enriched by DEGs of clusters 3 and 4. Translation-related genes were all the genes of GO term “translation” enriched by DEGs of cluster 6.

### Inference of Aging Lineage and Cell Pseudotime Along Lineages

In order to identify the continuous senescence of MEFs, we implemented Slingshot (Kelly et al., 2018) on the single-cell sequencing data. The cell clusters inferred by PCA and Louvain algorithm were used as input for Slingshot to infer the lineage relationship. Cluster 1 was set as the starting cells according to the expressing trend of aging genes we found in bulk RNA-seq data and previous studies. Pseudotime of each single cell along lineages was projected by principal curve implemented in Slingshot. Weight of projection was filtered by cutoff of 0.6. Lineage inference was performed on three panels of HVGs, which were around 2,500, 5,000, and 8,000, respectively. Three lineages, namely, the main lineage (1-2-3-4), duplication-regained lineage (1-2-5), and translation-elevated lineage (1-2-6), were supported by analysis using 2,500, 5,000, or 8,000 HVGs.

### Transcription Factor Network Discovery and Lineage Committed Transcription Factor Inference in the Main Lineage

We constructed co-expression modules between transcription factors (TFs) and candidate target genes (TGs). Motif rankings which indicate motif–target affinity were drawn from SCENIC database (Aibar et al., 2017). Co-expressed genes in the TF modules where treated as TF targets if they are enriched in the top motif rankings. Enrichment analysis of TF regulons in each single cell was implemented in each cell by calculating the AUC value of recovery curve. Pseudotime of each cell on certain lineage was inferred by principal curve implemented in Slingshot. Lineage committed TF networks were identified by examining the correlation between enrichment of TF network and the increase of cell pseudotime. AUC values of the recovery curve of each TF regulon were normalized across cells.

## Results

### Full-Length Single-Cell RNA Sequencing for Senescent Mouse Embryonic Fibroblasts

**FIGURE 1:**
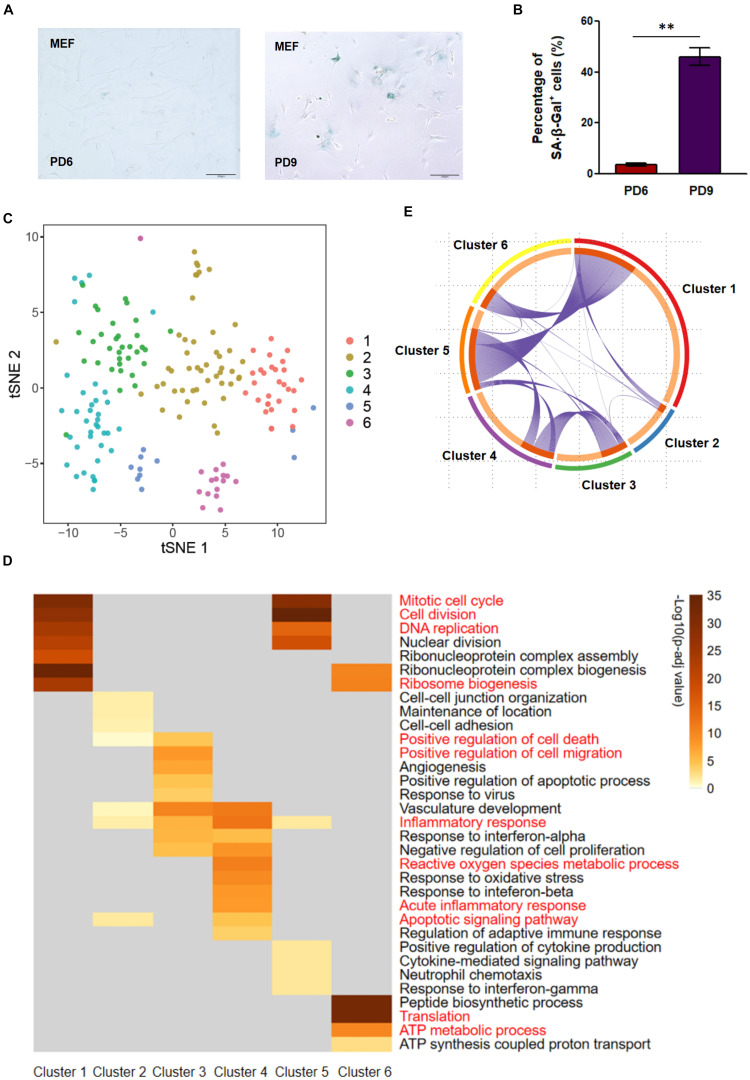
Single-cell RNA sequencing (RNA-seq) for senescent mouse embryonic fibroblasts (MEFs) revealed six clusters with distinct expression patterns. **(A,B)** Senescence-associated β-galactosidase (SA-β-Gal) staining of PD6 and PD9 MEFs. The left panel shows representative staining, and the right panel shows the quantitative evaluation. ^∗∗^*p*-value < 0.01, *t*-test. **(C)** T-distributed stochastic neighbor embedding (tSNE) visualization of six color-coded clusters of PD9 MEFs. **(D)** Heatmap of significantly enriched Gene Ontology (GO) terms using differentially expressed genes (DEGs) of each cluster. Clusters representing GO terms are highlighted in red color. **(E)** Cricos showing connections of DEGs of the six clusters. Purple lines link the identical DEGs between clusters. PD, population doubling.

To explore the heterogeneity and potential cell–cell interactions during replicative senescence of MEFs, we cultured MEFs in atmosphere (20%) oxygen condition until nearly half of the cells exhibited SA-β-Gal positive staining (Figures 1A,B). The advantage of this stage is that it includes both senescent and younger cells; thus, it is relatively cost-effective compared with select populations containing excesses of pro-senescent or senescent cells. Cell shape and viability were also evaluated prior to loading in the Fluidigm C1 system. Distinct differences among single cells were observed in terms of cellular shape (Supplementary Figure S1), suggesting that cells might undergo different stages of senescence process during a single passage. Single-cell transcriptome libraries were constructed in the C1 system and submitted to high-throughput sequencing. After quality control (including removal of potential contaminants and cell doublets), 175 single cells with an average sequencing depth of 3.96 million (M) reads per cell were selected for further analyses (Supplementary Figure S2). A total of 26,121 genes in total and an average of nearly 10,000 genes per cell were detected (Supplementary Figure S2), suggesting the high sensitivity with this full-length RNA-seq strategy. Subsequently, 2,556 genes that were more likely to reflect real biological differences among these single cells were selected based on gene expression dispersion, in accordance with previous publications (Macosko et al., 2015); these genes were subjected to further analysis (Supplementary Figure S3).

### Clustering Analysis Reveals Six Subpopulations of Mouse Embryonic Fibroblasts With Distinct Senescence Features

PCA coupled with clustering is widely used in single-cell transcriptome analysis (Zheng et al., 2017; Han et al., 2018). We thus applied it to our single-cell data; the 2,556 HVGs mentioned above were included in PCA. Because the top 50 PCs explained more than 90% of single-cell variance (Supplementary Figure S4), we chose them to cluster 175 MEFs by Seurat package (Macosko et al., 2015). One hundred seventy-five MEFs were split into six clusters and displayed in two-dimensional tSNE (Figure 1C and Supplementary Figure S5). DEG identification, followed by GO analysis, was carried out on these six clusters. Upregulated DEGs for each cluster were analyzed as compared to combined remaining five clusters to define the molecular features of this cluster. Overall, each cluster had characteristic GO terms (Figure 1D), reinforcing the reliability of the clustering analysis. Notably, several clusters also showed overlapped GO terms. For example, the senescence-associated GO term inflammatory response existed in both clusters 3 and 4 (Figure 1D), suggesting that these two clusters of cells were senescent but may have been at different stages of senescence.

We next analyzed these six clusters in a sequential manner. Cluster 1 was enriched in GO terms such as mitotic cell cycle, cell division, and DNA replication (Figure 1D); this suggested a normal replication status for the cells in the cluster. Cluster 2 had weak enrichment of normal function such as cell–cell junction organization and a few senescence-related GO terms such as inflammatory response (Figure 1D), implying that this group of cells was undergoing a transition state to senescence. Clusters 3 and 4 both showed signs of senescence (e.g., inflammatory response; Figure 1D). In addition, cluster 3 exhibited DEGs enriched in positive regulation of cell death and migration, while cluster 4 exhibited DEGs enriched in acute inflammatory response and apoptotic signaling pathway. As noted above, the results suggest that clusters 3 and 4 are at two different stages of senescence, as in previous reports where cell death-related genes were highly expressed in senescent cell populations (Kim et al., 2013). Notably, cluster 5 was a small but unique cluster in which upregulated DEGs were enriched in both cell duplication-associated GO terms (e.g., mitotic cell cycle and cell division) and SASP-associated GO terms (e.g., inflammatory response and positive regulation of cytokine production) (Figure 1D). There are two possible reasons to explain the appearance of these cells. One possibility is that these cells might be affected by SASP components in the culture, which were produced by adjacent senescent cells (e.g., cells in clusters 3 and 4) through a paracrine effect. The other possibility is that these cells might escape from cell cycle arrest (Sherr and Dipinho, 2000; Krones-Herzig et al., 2003). The exact origin of this small number of cells deserves further experimental validation. Cluster 6, another small but distinct cluster, exhibited OE of translation and ATP synthesis-associated GO terms based on enrichment analysis of upregulated DEGs (Figure 1D); this finding implied mass production of proteins coordinated with higher energy supply because protein translation is a biological process with a high energy demand (Lane and Martin, 2010; Gonskikh and Polacek, 2017). There were some shared GO terms between two clusters, such as mitotic cell cycle and cell division in both clusters 1 and 5, as well as ribosome biogenesis in both clusters 1 and 6. Thus, we examined whether these shared GO terms reflected similar or different DEGs in the same GO category. To systematically address this question, we analyzed all possible genes overlapping among these six clusters. We found that clusters 3 and 4 shared a considerable proportion of DEGs (Figure 1E). Certain DEGs in cluster 1 were also present in cluster 5 or 6 (Figure 1E), suggesting that these genes were dynamically regulated. Taken together, we defined these six clusters of single cells with signature genes and speculated that (1) cluster 1 was presumably proliferating cells that replicated normally; (2) cluster 2 could be an intermediate stage between clusters 3 and 4; (3) clusters 3 and 4 were presumably senescent cells with cell cycle arrest and SASP (Supplementary Figure S6); (4) cluster 5 represented a subset of MEFs that might be affected by SASP components in the culture or might escape from cell cycle arrest; (5) cluster 6 suggested that a subset of MEFs could coordinate protein translation with ATP synthesis.

**FIGURE 2:**
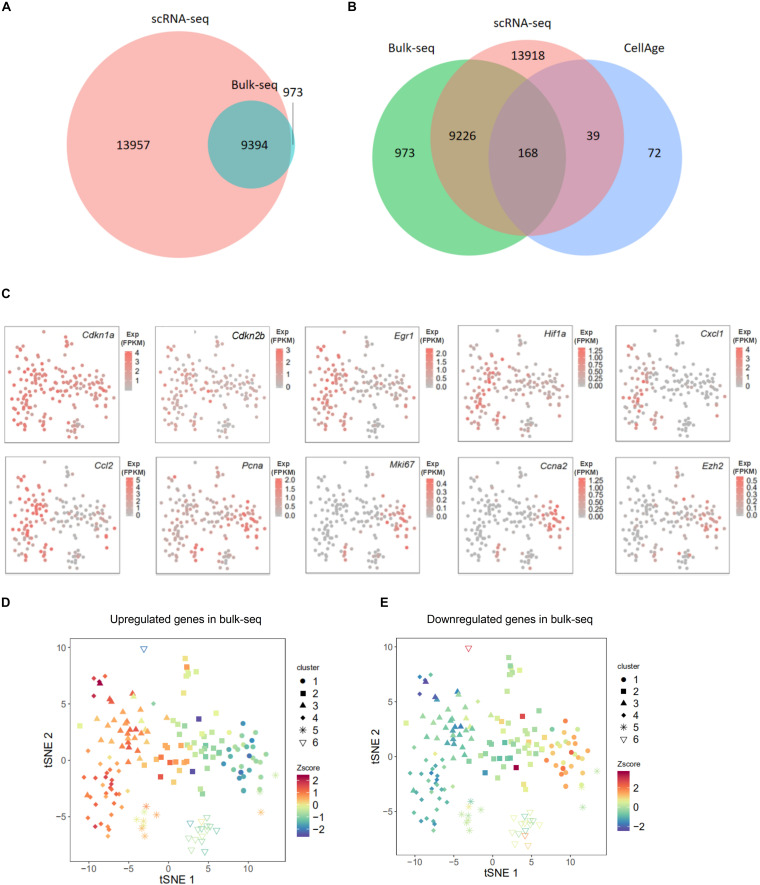
Comparison of single-cell RNA sequencing (scRNA-seq) data and bulk population data on mouse embryonic fibroblasts (MEFs). **(A)** Venn diagram presented the overlap of expressed genes between scRNA-seq data and our previous bulk population data on *in vitro* cultivated MEFs’ transcriptomes. **(B)** Venn diagram presented the overlap of expressed genes from scRNA-seq data, expressed genes from bulk population data, and senescence-associated genes from CellAge. **(C)** T-distributed stochastic neighbor embedding (tSNE) plots presenting the expression level of 10 well-known senescence markers in each single cell; these 10 genes were among the overlap of scRNA-seq data and bulk population data. **(D,E)** Overall expression levels of continuously upregulated **(D)** and downregulated **(E)** genes in our previous PD6 to PD11 MEF bulk population RNA-seq data in each single cell were presented in tSNE plot (Figure 1C). The overall expression level was displayed in normalized area under the curve (AUC) level (Z-score). Z-score, value calculated by Z normalization using AUC value; Bulk-seq, PD6 to PD11 MEF bulk population RNA-seq; Exp, expression level.

To further confirm the senescence status of each cluster, we applied our previously published bulk population MEF RNA-seq data (Chen et al., 2018), which contain transcriptomes of *in vitro*-cultivated MEFs from PD6 to PD11 to the current single-cell data. As expected, two data sets shared a large proportion of expressed genes (Figure 2A). To further investigate shared genes between two data sets, we overlapped those shared genes with literature-supported senescence-associated genes (from CellAge^1^). Approximately 60% (168 out of 279) senescence-associated genes were simultaneously expressed in two data sets (Figure 2B). Among expressed genes that were shared in these two datasets, we individually displayed expression levels of a subset of well-known senescence marker genes in the tSNE map. Cyclin-dependent kinase inhibitor (CKDI) coding genes like *Cdkn1a* and *Cdkn2b* had relatively higher expression levels in clusters 3 and 4 than in cluster 1 (Figure 2C), as did the senescence-associated TF coding gene *Egr1*, SASP-related genes like *Cxcl1* and *Ccl2* (Figure 2C). In contrast, the cell proliferation marker gene *Mki67* showed a higher expression level in cluster 1 than in clusters 3 and 4. We also performed expression plots of gene pairs (*Cdkn1a* and *Cdkn2b*, *Cdkn1a* and *Egr1*, *Cdkn1a* and *Hif1a, Cdkn1a* and *Pcna*) to further explore the co-expression at the single-cell level. Expression levels of *Cdkn1a* and *Cdkn2b*, expression levels of *Cdkn1a* and *Egr1*, and expression levels of *Cdkn1a* and *Hif1a* were positively correlated. Although expression levels of *Cdkn1a* and *Pcna* were not negatively correlated, the expression level of *Cdkn1a* in most cells was higher than 1 FPKM, whereas the expression level of *Pcna* in most cells was lower than 1 FPKM (Supplementary Figure S7). The result above suggested that cluster 1 exhibited transcriptome features of pro-senescent cells, whereas clusters 3 and 4 exhibited transcriptome features of senescent cells.

Next, we selected the genes that were consecutively upregulated (1,531 genes) or downregulated (1,993 genes) during the course of *in vitro* cellular senescence in MEFs (from PD6 to PD11; Chen et al., 2018) and displayed their overall expression level (displayed by Z score) in the two-dimensional tSNE map of single cells (Figures 2D,E). As expected, upregulated genes in bulk population data demonstrated higher expression levels in clusters 3 and 4 (Figure 2D), in which DEGs were enriched in senescence-associated GO terms. Similarly, downregulated genes in bulk population data demonstrated high expression levels in cluster 1 (Figure 2E), in which DEGs were enriched in duplication-associated GO terms. Cells from cluster 2 expressed subsets of both upregulated and downregulated genes (Figures 2D,E). This result further supported that MEFs in cluster 1 were younger than that in clusters 3 and 4; cluster 2 exhibited intermediate features. To further illustrate this point, we plotted the expression of literature-supported senescence-associated genes (from CellAge^1^, within those consecutively upregulated or downregulated genes on the two-dimensional tSNE map. The conclusion remained consistent with the finding above (Supplementary Figure S8). Together, we provided three types of evidence (including GO analysis of DEGs for each cluster, classical senescence marker genes, and bulk RNA-seq from senescent MEFs) to support the notion that these six clusters reflect distinct stages of MEF senescence.

### Trajectory Analysis Reveals Three Distinct Lineages During Mouse Embryonic Fibroblast Senescence

**FIGURE 3:**
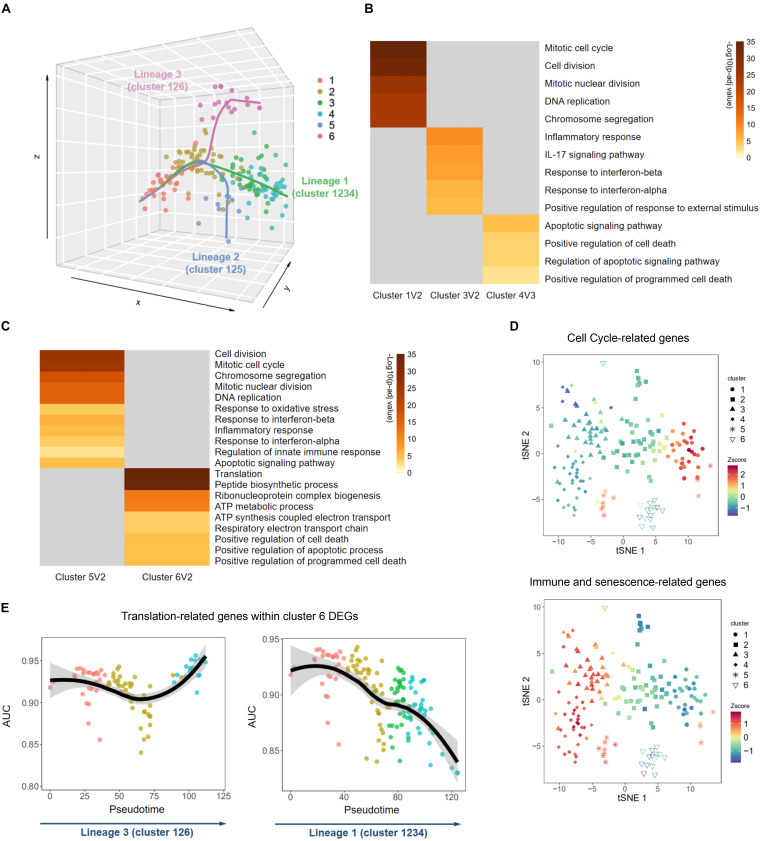
Distinct biological processes of senescence uncovered by trajectory inference. **(A)** Trajectory analysis by Slingshot revealed three development lineages during senescence. **(B,C)** Heatmaps of main Gene Ontology (GO) terms enriched in Metascape using differentially expressed genes (DEGs) between two adjacent clusters in lineage 1 **(B)** and lineages 2 and 3 **(C)**. Only highly expressed DEGs were used for GO terms enrichment. **(D)** T-distributed stochastic neighbor embedding (tSNE) plots of overall expression level of cell cycle-related genes (upper panel) and immune and senescence-related genes (lower panel) in each single cell. **(E)** Graphs showing overall expression levels of translation-related genes selected from cluster 6 DEGs in each single cell along the pseudotimes of lineages 3 (left panel) and lineage 1 (right panel). Z-score, value calculated by Z normalization using the AUC value.

The above results indicated that MEFs were at different stages of senescence, although they had been cultured simultaneously. Different clusters might reflect different senescence statuses of MEFs through the senescence paths. To explore whether such paths exist, we analyzed the development trajectories of single-cell transcriptome to investigate relationships among these six clusters. Along the pseudotime, there were three indicated lineages, all of which began with cluster 1, followed by cluster 2, and then diverged into three different branches (Figure 3A and Supplementary Figure S9). This result suggested that MEFs might undergo different biological processes during cellular senescence. The main lineage contained clusters 1, 2, 3, and 4, which possessed a large proportion of all single cells and could be a major senescence path. Compared with cluster 2, cluster 1 exhibited upregulation of DEGs that were enriched in duplication-associated GO terms, while cluster 3 exhibited upregulation of DEGs that were enriched in senescence-associated GO terms (Figure 3B); these findings suggested that cluster 1 was younger than cluster 2, while cluster 3 exhibited greater senescence than cluster 2. Next, when conducting DEG analysis between clusters 3 and 4, we found that GO terms such as apoptotic signaling pathway and positive regulation of cell death were exclusively displayed in cluster 4, consistent with the hypothesis that apoptosis and cell death is the next step of senescence (Figure 3B; Kim et al., 2013). These results implied that MEFs in this lineage first underwent cell cycle arrest (from cluster 1 to cluster 2), and then acquired SASP and inflammation-related traits (from cluster 2 to cluster 3), and finally underwent apoptosis or cell death (from cluster 3 to cluster 4). This lineage was also supported by the finding of a previous study that showed senescent human diploid fibroblasts (HDFs, another commonly used cell line in studying senescence) first exhibited features of reduced cell cycle-related genes, followed by elevated expression of inflammation and apoptosis-associated genes and ultimate elevation of cell death-related genes (Kim et al., 2013). The second lineage contained clusters 1, 2, and 5. To understand this lineage further, we conducted DEG analysis between clusters 5 and 2. The result revealed that upregulated DEGs in cluster 5 were enriched in both cell duplication-associated GO terms and senescence-associated GO terms, suggesting a possibility of escape from cell cycle arrest and acquisition of SASP for cells in cluster 5 (Figure 3C). Furthermore, we selected cell cycle-related genes described in a previous publication (Macosko et al., 2015), as well as immune and senescence genes from clusters 3 and 4 DEGs and plotted them on the clusters. We found that cells in cluster 5 expressed higher levels of both cell cycle and immune, senescence-related genes (Figure 3D), further supporting the distinct status of this cluster. Previous studies demonstrated that a small amount of *in vitro* cultured MEFs may be immortal because of mutations in the p53-MDM2-p19ARF pathway (Krones-Herzig et al., 2003). That might explain how cells in cluster 5 were able to escape cell cycle arrest. The third lineage contained clusters 1, 2, and 6. When compared with cluster 2, cluster 6 did not demonstrate an upregulation of inflammation-related genes, instead upregulated DEGs were enriched in translation and ATP synthesis-associated GO terms (Figure 3C), suggesting coordinated expression of these two biologically relevant gene types. Intriguingly, the overall downregulation of translation-related ribosomal proteins in the main lineage (1-2-3-4) was not detected in this lineage (1-2-6) (Figure 3E). A previous study also observed that ribosomal protein levels and overall translation were elevated in premature aging (Buchwalter and Hetzer, 2017). The underlying mechanism by which enhanced translation and ATP synthesis occurred during *in vitro* cultivation of MEFs is unclear and merits further investigation. Together, trajectory analysis of these single cells revealed three distinct lineages of MEFs during cellular senescence, namely, the main lineage (1-2-3-4), duplication-regained lineage (1-2-5), and translation-elevated lineage (1-2-6).

### Upregulation of Some Senescence-Associated Secretory Phenotype Components Was Observed in Subsets of Cells During Senescence of Mouse Embryonic Fibroblasts Cultivated in Atmospheric Oxygen

**FIGURE 4:**
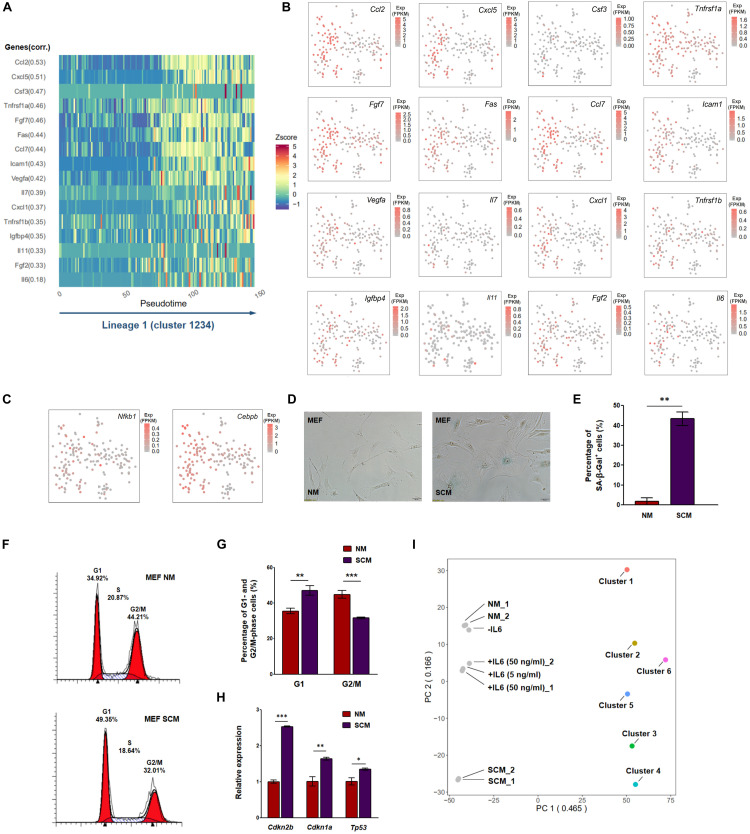
Upregulation of some senescence-associated secretory phenotypes (SASPs) was observed in atmospheric oxygen-cultivated mouse embryonic fibroblasts (MEFs). **(A)** Representative SASP components were upregulated along lineage 1 in Figure 3A. Z-score, value calculated by Z normalization using expression level; corr, correlation score along lineage 1. **(B,C)** T-distributed stochastic neighbor embedding (tSNE) plots presenting expression levels of upregulated SASPs **(B)** and their upstream transcription factors (TFs) **(C)** in each single cell. As shown in the picture, SASPs and two SASP-related TFs were upregulated in clusters 3 and 4 in a scattered matter. **(D–H)** Senescence-associated β-galactosidase (SA-β-Gal) staining **(D)** and its quantitative evaluation **(E)**, cell cycle analysis **(F)** and its quantitative evaluation **(G)**, and quantitative reverse transcription PCR (qRT-PCR) of several senescence markers **(H)** were compared between normal medium (NM)- and senescence-conditioned medium (SCM)-cultivated MEFs. *Gapdh* was used as internal control in qRT-PCR. *, **, and *** represent *p*-values less than 0.05, 0.01, and 0.001 by *t*-test, respectively. **(I)** Cells cultivated with NM, SCM, medium without interleukin (IL)6, medium with 5 ng/ml IL6, and medium with 50 ng/ml IL6 were collected and subjected to RNA sequencing (RNA-seq). Principal component analysis (PCA) was performed with bulk RNA-seq data and single-cell RNA-seq data (cells in the same cluster were combined together) using their overlapped genes. PC1 likely represents the data type difference between bulk and single-cell RNA-seq. NM, normal medium-cultivated MEFs; SCM, senescence-conditioned medium-cultivated MEFs; -IL6, MEFs cultivated in medium without IL6; + IL6 (5 ng/ml)/ + IL6 (50 ng/ml), MEFs cultivated in medium with 5 or 50 ng/ml IL6. Two replicates (_1 and _2) of samples with NM, SCM, and 50 ng/ml IL6 were included for analysis. Exp, expression level.

Factors that contributed to the main lineage (1-2-3-4) were next examined. We noticed that some SASP components were upregulated as cells progressed through this lineage (Figure 4A). However, only a subset of cells exhibited upregulation of these factors (Figure 4B), whereas other cells did not change. Notably, *Nfkb1* (p50) and *Cebpb*, two well-known TF coding genes that have been reported to regulate SASP (Freund et al., 2010), were also upregulated in a subset of cells in the lineage 1-2-3-4 (Figure 4C), implying that certain TF-SASP signal signaling interactions contribute to senescence of some cells in this lineage. This result was initially considered contradictory to the findings of a previous study, which reported that the secretion of SASP factors from atmospheric oxygen cultivated MEFs was not detected during the progression of senescence (Coppé et al., 2010). However, because the discovered SASP-related genes such as *Il6* and *Cxcl1* were only upregulated in a small percentage of cells (Figures 4A,B), those genes presumably could not be detected by bulk cell analysis due to a dilution effect, whereas they could be detected by single-cell analysis. To further confirm the existence of SASP in MEFs, we simultaneously cultivated MEFs with NM and SCM collected from senescent MEFs. Enhancement of SA-β-Gal staining and G1-phase cell cycle arrest was observed in SCM-cultivated MEFs compared with NM-cultivated cells (Figures 4D–G). Additionally, upregulation of *Cdkn2b* and *Cdkn1a* was observed in SCM-cultivated cells by reverse transcription followed by qRT-PCR (Figure 4H). Because SCM contains SASP mediators such as IL6 that are released by senescent cells (Coppé et al., 2010), the above results support the notion that atmospheric oxygen-cultivated MEFs can be induced to senescence through an SASP mechanism.

To explore if a single SASP factor such as IL6, for which expression was upregulated in a few of the senescent MEFs, can lead to senescence by means of paracrine signaling, we added IL6 into culture medium and examined senescence-associated phenotypes in MEFs by comparison with control medium. The impact of IL6 was less striking than the addition of SCM, which contains multiple SASP factors, however, a paracrine tendency was observed, including a higher percentage of SA-β-Gal staining and elevated *Cdkn2b* expression upon IL6 treatment in younger MEFs (Supplementary Figure S10). This supported the notion that a few MEFs could secrete SASP mediators such as IL6 to promote senescence of nearby cells in a paracrine manner. To examine the similarity of cellular senescence induced by SCM (or IL6) and the senescent path in the main lineage, we performed bulk population RNA-seq on MEFs with and without SCM or IL6 treatment. PCA was applied to compare bulk RNA-seq with single-cell clusters. Because PC1 apparently differed on the basis of RNA-seq data type (bulk vs. single cell), we used PC2 for comparison of real biological differences. Consistent with the enhanced SA-β-Gal staining upon SCM treatment, we found that SCM-cultivated MEFs were close to clusters 3 and 4, while NM-cultivated cells were close to clusters 1 and 2 with respect to whole transcriptome expression (Figure 4D). Consistent with the weak impact observed regarding *Cdkn2b* (Figure 4H and Supplementary Figure S10), IL6-treated MEFs also demonstrated a moderate transcriptome shift to senescence compared with NM treatment (Figure 4I). Overall, the above data showed that some SASP components were upregulated in a subset of cells during senescence of atmospheric oxygen-cultivated MEFs, which might be related to paracrine senescence.

### Network Analysis Shows HOXD8 Is a Novel Regulator for Mouse Embryonic Fibroblast Senescence in the Main Lineage

**FIGURE 5:**
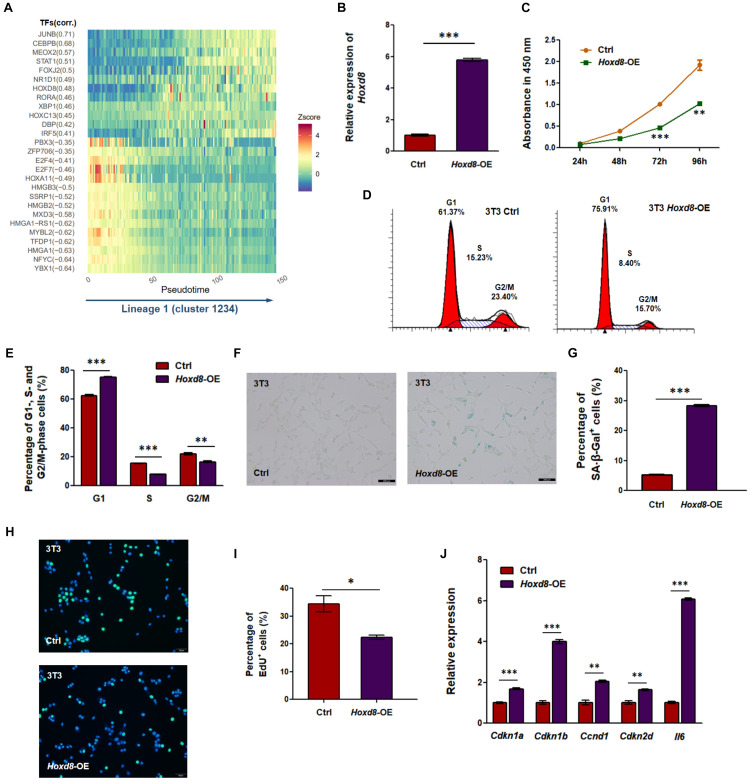
Transcription factor (TF) HOXD8 is a new senescence regulator in mouse embryonic fibroblasts (MEFs). **(A)** Co-expression modules of TFs and their predicted target genes (TGs) were filtered using TF-motif database in RcisTarget along lineage 1 (Aibar et al., 2017). Pearson correlation was calculated between the overall expression of co-expression genes and lineage 1 pseudotime. Modules with correlation coefficient (corr.) either more than 0.3 or less than -0.3 are displayed in heatmap. Z-score, value calculated by Z normalization using area under the curve (AUC) value. **(B)** Overexpression (OE) of Hoxd8 in NIH3T3 cells was confirmed by quantitative reverse transcription PCR (qRT-PCR). *Gapdh* was used as internal control. **(C)** Cell proliferation rate was evaluated by Cell Counting Kit-8 (CCK-8) assay. **(D,E)** Cell cycle analysis between Hoxd8-OE and control NIH3T3 cells. **(D)** Shows representative cell cycle distribution, and **(E)** shows the quantitative evaluation. **(F,G)** Senescence-associated β-galactosidase (SA-β-Gal) staining **(F)** and its quantitative evaluation **(G)** were compared between Hoxd8-OE and control NIH3T3 cells. **(H,I)** 5-Ethynyl-2′-deoxyuridine (EdU) incorporation assay **(H)** and its quantitative evaluation **(I)** were compared between Hoxd8-OE and control NIH3T3 cells. Blue, 4′,6-diamidino-2-phenylindole (DAPI) staining; green, EdU incorporation. **(J)** qRT-PCR analysis for cell cycle and senescence-related genes in both Hoxd8-OE and control cells. *Gapdh* was used as internal control. ^∗^, ^∗∗^, and ^∗∗∗^ represent *p*-values less than 0.05, 0.01, and 0.001 by *t*-test, respectively.

We hypothesized that dynamically changed TF may regulate multiple TGs including SASP to promote senescence in the main lineage. To screen potential TF–TG networks, we selected and filtered TFs and their co-expression genes using a pipeline similar to SCENIC (Aibar et al., 2017). We identified lineage-committed TF–TG networks on the basis of the correlation between enrichment of TGs in cells (measured by AUC of recovery curve of the TGs) and cell pseudotime of the main lineage inferred by Slingshot (correlation > 0.3 or < -0.3; Figure 5A). Notably, several TFs (e.g., JUNB, CEBPB, MEOX2, and TFDP) have been shown to inhibit cell proliferation and induce cellular senescence (Konishi et al., 2008; Douville et al., 2011; Ohdaira et al., 2012; Thomsen et al., 2015), suggesting that these discovered TF–TG networks reliably contributes to senescence. To further investigate TFs that have not been identified as regulators of cellular senescence, we overexpressed *Nr1d1* and *Hoxd8*, for which corresponding TGs exhibited positive correlations with cell pseudotime, and knocked down *Ssrp1* and *Nfyc*, for which corresponding TGs exhibited negative correlations with cell pseudotime (Figure 5A and Supplementary Figure S11). We discovered that OE of *Hoxd8* could promote senescence-associated phenotypes (Figures 5B–J). HOXD8 is a TF that has been reported to regulate cell proliferation and differentiation and to determine regional identity during embryogenesis (Mcginnis and Krumlauf, 1992; Liu et al., 2016; Mansour and Senga, 2017). OE of *Hoxd8* in NIH3T3 (Figure 5B), a mouse fibroblast cell line derived from MEFs, led to multiple senescence-associated phenotypes, including reduced cell proliferation rate (Figure 5C), G1 phase arrest (Figures 5D,E), enhanced SA-β-Gal staining (Figures 5F,G), and reduced DNA replication activity (Figures 5H,I). Upregulation of senescence markers including *Cdkn1a*, *Cdkn1b*, *Ccnd1*, and *Ckdn2d* was also observed by qRT-PCR (Figure 5J). Notably, the expression of *Il6* was also enhanced in *Hoxd8*-OE cells (Figure 5J). Transcriptomic analysis revealed DEGs between *Hoxd8*-OE and normal NIH3T3 cells that were enriched in senescence-related GO terms such as cellular response to DNA damage stimulus, cell division, and regulation of translation (Supplementary Figure S12). We also observed a tendency of cellular senescence in *Hoxd8*-OE MEFs (Supplementary Figure S13). These results indicate that HOXD8 could be a novel regulator in regulating MEF senescence, according to both single-cell analysis and experimental validation.

## Discussion

To the best of our knowledge, this study is the first single-cell transcriptome analysis of replicative senescent MEFs, a basic and valuable cellular aging model. Although it has been known that senescent cells are not identical, even at the same passage during *in vitro* cultivation, there has been no clear understanding of this heterogeneity among single MEFs. By sequencing each single cell at a half SA-β-Gal staining passage of MEFs with sufficient sequencing coverage, we identified dramatic differences among these single cells. Six clusters were discovered, and each represents a distinct status with signature genes associated with different stages of MEF senescence. Furthermore, these six clusters reflected three lineages, indicating three distinct cell fates that each single cell may choose. Whereas a previous bulk population study suggested that senescent MEFs cultivated in atmospheric oxygen did not secrete higher levels of any SASP factors, compared with pro-senescent MEFs (Coppé et al., 2010), our single-cell transcriptome analysis revealed that at least a few MEFs exhibited a higher expression of SASPs and their upstream TFs. By integrative analysis and experimental validation, we also identified the TF HOXD8 as a novel regulator for MEF senescence. Thus, this study thus provides a new perspective for this important senescence model.

A previous study performed extensive analyses of the transcriptome dynamics of HDFs over the course of *in vitro* replicative senescence, however, the heterogeneity of these senescent cells remained unclear (Kim et al., 2013). Using single-cell RNA-seq, we detected three distinct lineages in replicative MEFs. The main lineage manifested features on SASPs, inflammation, and cell death, similar to the pattern of HDFs (Kim et al., 2013). However, the duplication-regained lineage indicated a possibility of recovery of cell replication ability and persistent expression of SASPs; the translation-elevated lineage showed a coupled enhancement of translation and ATP synthesis. These two lineages only comprised a small percentage of single cells and may be overwhelmed by bulk transcriptome analysis. Thus, single-cell analysis of MEFs provided us a complex but intriguing perspective of gene expression in senescence. It revealed that single cells may choose different cell fates and exhibit distinct gene expression features upon exposure to replicative stress. Notably, the relationship between global translation level and cellular senescence/aging has been reported in other studies with various study materials. A previous study demonstrated enhancement of global translation level and ribosome synthesis in a premature aging mouse model (Buchwalter and Hetzer, 2017). Additionally, multiple studies showed life span extension in flies or worms in knockout mutants with certain ribosomal protein genes (Steffen et al., 2008; Janssens and Veenhoff, 2016). Because cellular senescence is known to promote individual aging (López-Otín et al., 2013), the higher translation activity in cluster 6, compared with cluster 2, suggested a possible link between protein synthesis and senescence, which merits further investigation.

To deeper understand the driving force of senescence in the main lineage, we investigated the SASP components. Some SASPs were detected to be upregulated in the senescent clusters in a scattered pattern (only upregulated in some cells). A previous study did not detect significantly higher secretion levels of any SASP factor in senescent MEFs (e.g., cultivated in atmospheric oxygen) compared with pro-senescent MEFs (Coppé et al., 2010). This disparity might be related to differences in the experimental approach between these two studies; in particular, upregulation of SASPs in a small proportion of cells might not change the overall expression level. That is another insight that can only be observed by single cell-based experiments. Consistent with the findings of previous studies (Hubackova et al., 2012; Acosta et al., 2013), we observed the induction of paracrine senescence in MEFs by SASP components when cells were exposed to conditioned medium; IL6-treated MEFs displayed weaker senescence features compared with SCM-cultivated MEFs.

*Hoxd8* belongs to the *Hox* gene family, an important regulator family that functions in embryogenesis (Mcginnis and Krumlauf, 1992). Recent studies suggested that reactivation of this developmentally critical gene family played an unexpected role during aging (Schwörer et al., 2016). For example, *Hoxa9* was discovered to be reactivated in aged individuals and impaired the function of the muscle stem cell (Schwörer et al., 2016). As for *Hoxd8*, it has been reported to regulate the cell cycle and oncogenesis in several carcinomas (Liu et al., 2016; Mansour and Senga, 2017). By single-cell analysis of MEFs, we first showed that *Hoxd8* could also be a regulator of senescence, extending the link between this gene family and aging. ChIP-seq coupled with RNA-seq analysis need to be performed in a future investigation to fully elucidate the direct TGs of this TF with respect to senescence.

Zirkel et al. (2018) carried out scRNA-seq on ∼8,300 proliferating and ∼5,200 replicative senescent HUVECs on a 10X Genomics platform. We found multiple senescent marker genes [*CDKN1A* (p21), *PCNA*, *LMNB1*, *EZH2*, *CCNA2*, *IL6*] had similar heterogeneity between replicative MEFs and HUVECs (Figures 2B,D from Zirkel et al., 2018, and Figure 2C from our study). For example, *PCNA* showed higher expression in proliferating cells while reduced expression in senescent cells (in both MEFs and HUVECs). IL6 exhibited increased cell number of IL6^high^ cells in senescent HUVECs compared with younger cells, but the overall cell proportion is not very high (Figure 2B in Zirkel et al., 2018), consistent with our finding in replicative MEFs that a few of the senescent MEFs had high expression of *Il6* (Figure 4B). *CDKN1A* showed an increased expression trend along the pseudotime course of HUVECs (Figure 2D from Zirkel et al., 2018), in line with the elevated expression trend of *Cdkn1a* along the main lineage (1-2-3-4) of MEFs (Figure 2C, Figure 3). In Figure 3 of our MEF data, we showed that immune and senescence-related genes had an increased expression trend along the main lineage. However, SASP-related genes such as *CXCL1* and *IL8* did not show an increased expression trend along the pseudotime course of HUVECs (Figure 2D from Zirkel et al., 2018). One possible explanation is that Zirkel et al. (2018) sequenced senescence entry cells while some of the MEFs we sequenced were at a later stage of senescence, which is featured by immune-senescence cross talk-related genes. Since the study of Zirkel et al. (2018) focused on the function and mechanism of candidate gene *HMGB2*, after discovering the eight senescent cell states along the main path, they did not perform further analysis on scRNA-seq data. We thus did not know the dynamic changes of translation-related genes in HUVECs, and this point deserves further study.

Teo et al. (2019) performed scRNA-seq (Smart-Seq2 protocol) in an H-RasG12V-induced IMR90 fibroblast model. They obtained transcriptome data of hundreds of single cells. The main discovery of their study is that two cell senescence endpoints were identified, and the primary endpoint is featured by Ras while the secondary one is marked by Notch signaling activation. Interestingly, cell cycle-related genes such as *CDKN1A* and *CDKN2B* showed distinct expression profiling in these two different senescence states (Figure 1D from Teo et al., 2019). In our MEF model, we also observed dynamic changes of cell cycle-related genes along the three lineages identified (Figures 2C, Figures 3A,D). One common feature between replicative MEFs and oncogene-induced IMR90 senescence is elevated expression of *CEBPB*. We found increased *Cebpb* along the main lineage of senescent MEFs (Figures 4A,C, Figure 5A), and Teo et al. (2019) also discovered upregulated *CEBPB* in both primary and secondary senescent states in IMR90 (see Figure 2D from Teo et al., 2019). We also found that a few SASP genes such as CXCL and IL family members showed a similar upregulation in senescent MEFs and IMR90 cells (Figures 1D, H from Teo et al., 2019; Figures 2–4).

There were some limitations in our study. We observed different senescence stages of single cells from PD9 MEFs and reconstructed the senescence process by lineage analysis. It would be preferable to chronologically sequence the single-cell transcriptomes of cells from the first *in vitro* cultivation passage to the last passage, which would allow construction of the authentic temporal lineage of each cluster during cell passages. That will help us understand more about the mechanisms involved in senescence (for example, when does the cluster with upregulated translation genes and ATP synthesis genes appear, and whether it will disappear in the end). Another limit is that we provided some new insights about how paracrine effects and *Hoxd8* functioned in the senescence process of mouse fibroblasts, but we still did not investigate whether and how they function in other replicative senescence models, premature aging models, or aged tissues/organisms. Future investigations following this study are warranted to fully understand the whole picture of senescence at the single-cell level.

## Data Availability Statement

The datasets presented in this study can be found in online repositories. The names of the repository/repositories and accession number(s) can be found at: https://bigd.big.ac.cn/gsa/browse/CRA002582, CRA002582.

## Author Contributions

WC conducted the single-cell RNA-seq and cellular experiments and drafted the manuscript. PQ, XW, and GW analyzed the data. YH and DL directed the experiments of single-cell RNA-seq. YS provided the support of Fluidigm C1 system. SQ and JC conducted the partial cellular experiments. BZ separated the MEFs from mouse. MC directed the cellular experiments. WJ directed the data analysis. TN designed the project, directed result analysis, and revised the manuscript. All authors contributed to the article and approved the submitted version.

## Conflict of Interest

DL was employed by the Fluidigm (Shanghai) Instrument Technology Co., Ltd. The remaining authors declare that the research was conducted in the absence of any commercial or financial relationships that could be construed as a potential conflict of interest.

## Footnotes

1 http://genomics.senescence.info/cells/
