# Supplementary material for: Single-Cell Transcriptome Analysis Reveals Six Subpopulations Reflecting Distinct Cellular Fates in Senescent Mouse Embryonic Fibroblasts
Source: Front Genet. 2020 Aug 11;11:867. doi: 10.3389/fgene.2020.00867 (PMC7431633; doi:10.3389/fgene.2020.00867)
Supplement: Supplementary file 1 [file Image_1.pdf]

Supplementary Figures

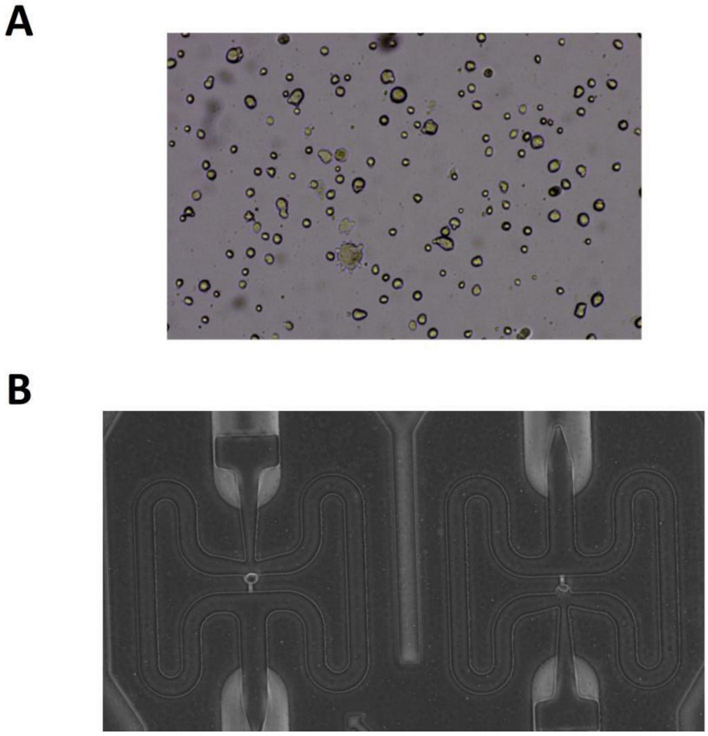

Fig s1. Single cell separation evaluation by Fluidigm C1 system. (A) Microscope view of single cells obtained from PD9 MEFs before loading to C1 system. (B) Single cells were captured in C1 Single-Cell mRNA Seq IFCchip (Fluidigm). Imaged on an inverted microscope.

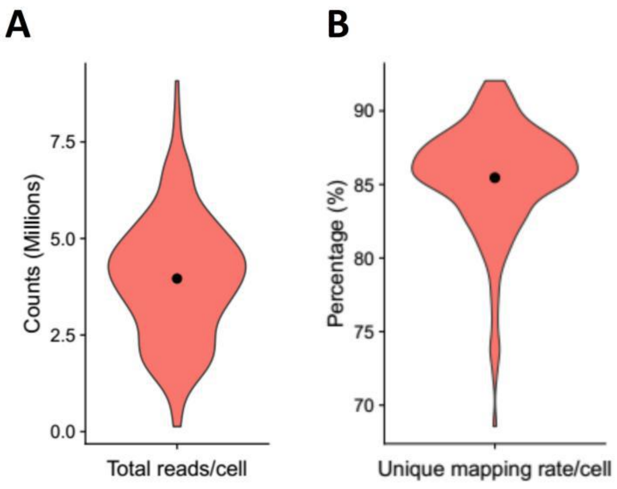

Fig s2. Quality control of scRNA-seq data of MEFs. Total reads (A), unique mapping ratio (B) and genes detected (C) per single cell were shown in violin plot.

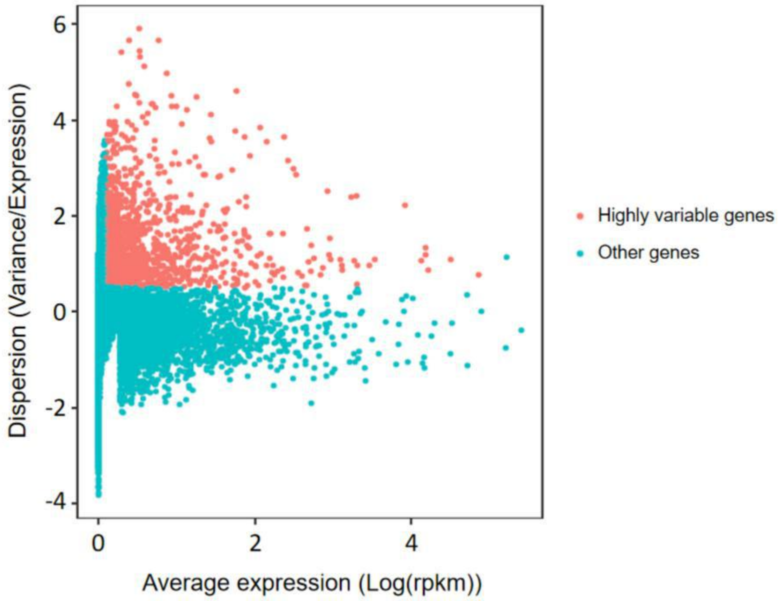

Fig s3. 2556 highly variable genes were selected using the method provided in Macosko et al., 2015.

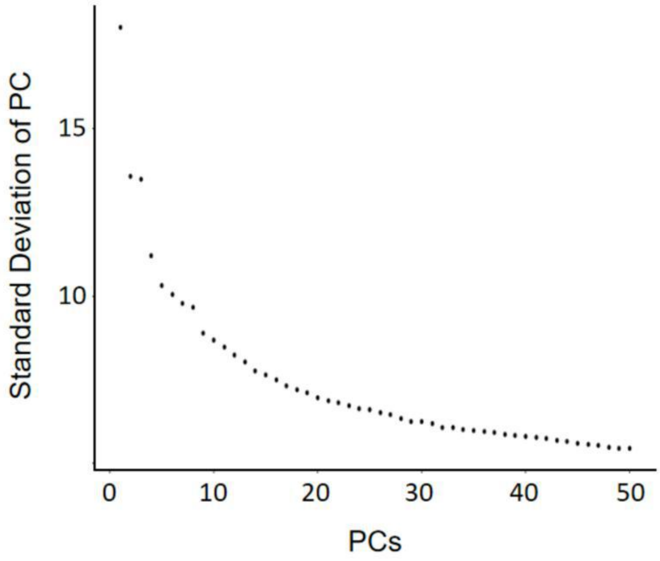

Fig s4. Top 50 PCs and their explanation of single cell transcriptome variance.

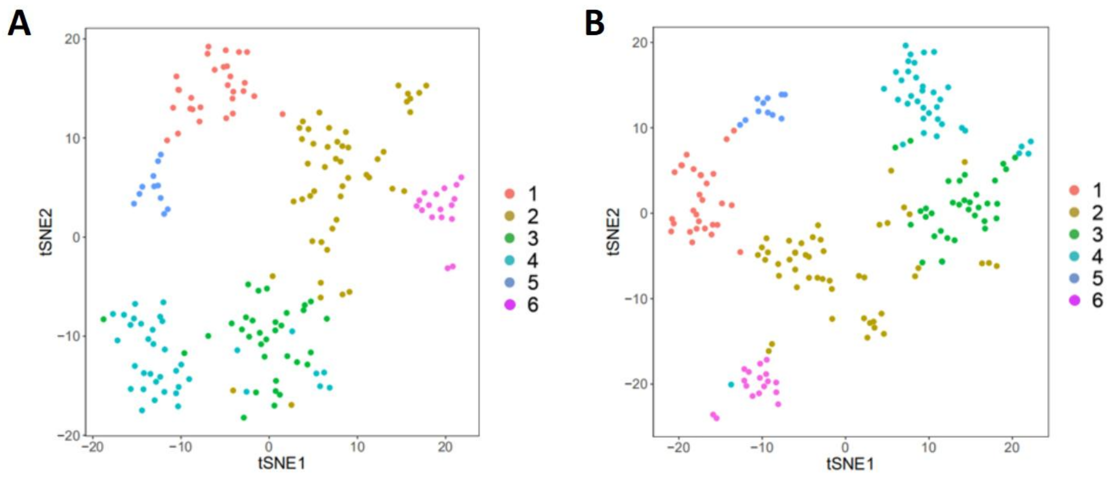

Fig s5. tSNE plot displaying clustering results using 5000 (A) or 8000 (B) highly variable genes. For comparison, we colored the dots in this figure using the clustering information obtained from 2556 highly variable genes (Figure 1C); cells that belong to the same cluster in Figure 1C still gathered together when using 5000 or 8000 genes for clustering, suggesting the overall agreement by using different number of highly variable genes.

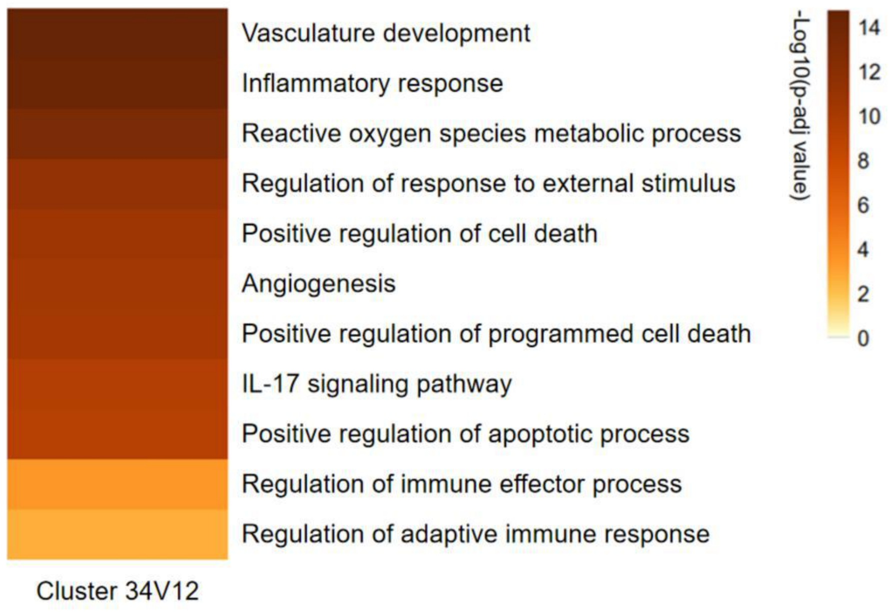

Fig s6. Heatmaps of GO terms enriched in Metascape using DEGs from cluster 3 and 4 versus cluster 1 and 2. Only highly expressed DEGs were used for GO terms enrichment.

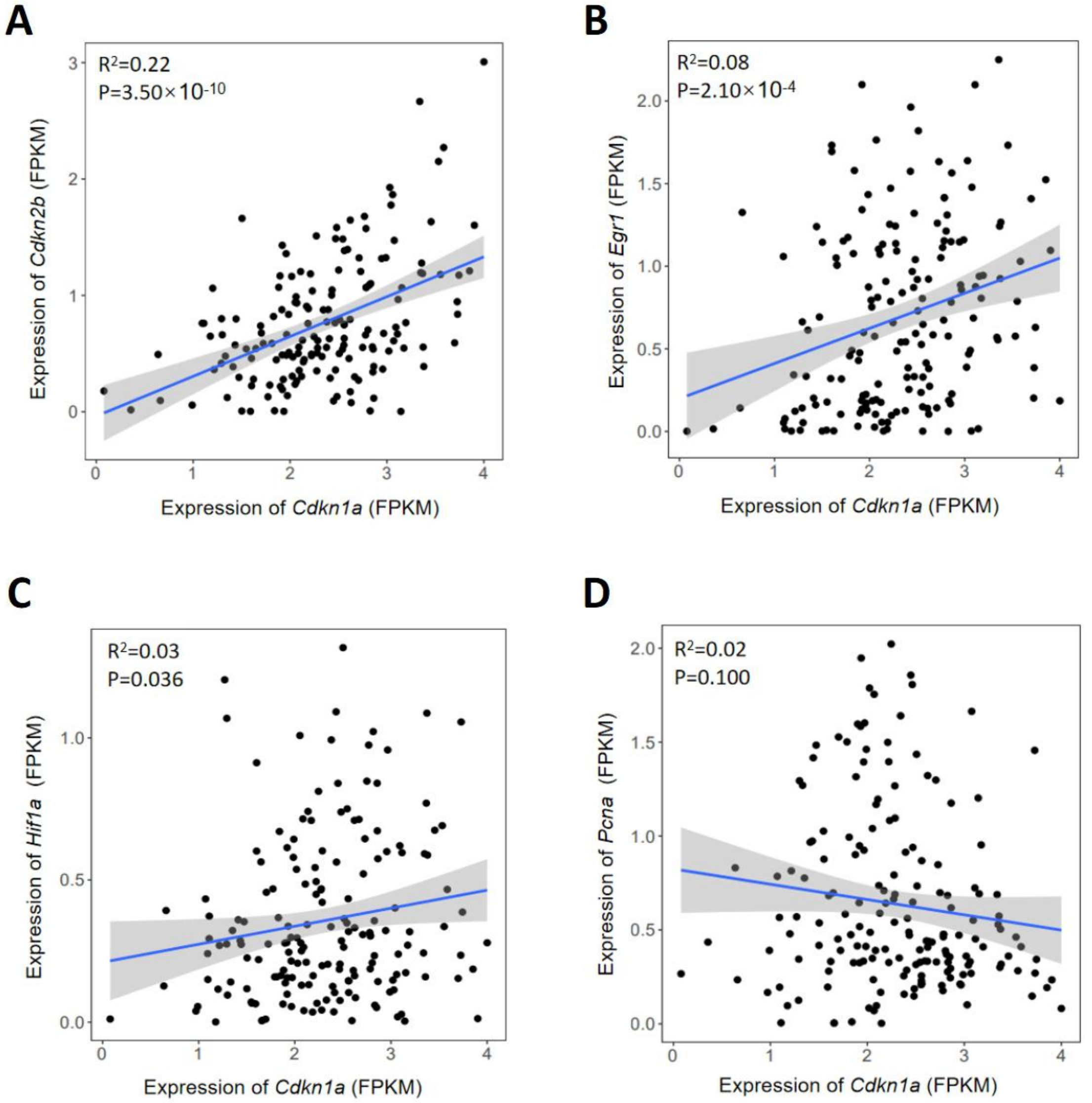

Fig s7. Comparison of senescence marker genes shared in scRNA-seq and bulk population MEF RNA-seq data (Chen et al., 2018). A. Comparison of expression levels of *Cdkn1a* and *Cdkn2b*; B. Comparison of expression levels of *Cdkn1a* and *Egr1*; C. Comparison of expression levels of *Cdkn1a* and *Hif1a*; D. Comparison of expression levels of *Cdkn1a* and *Pcna*. Grey areas represent variance. FPKM: Fragments Per Kilobase Million.

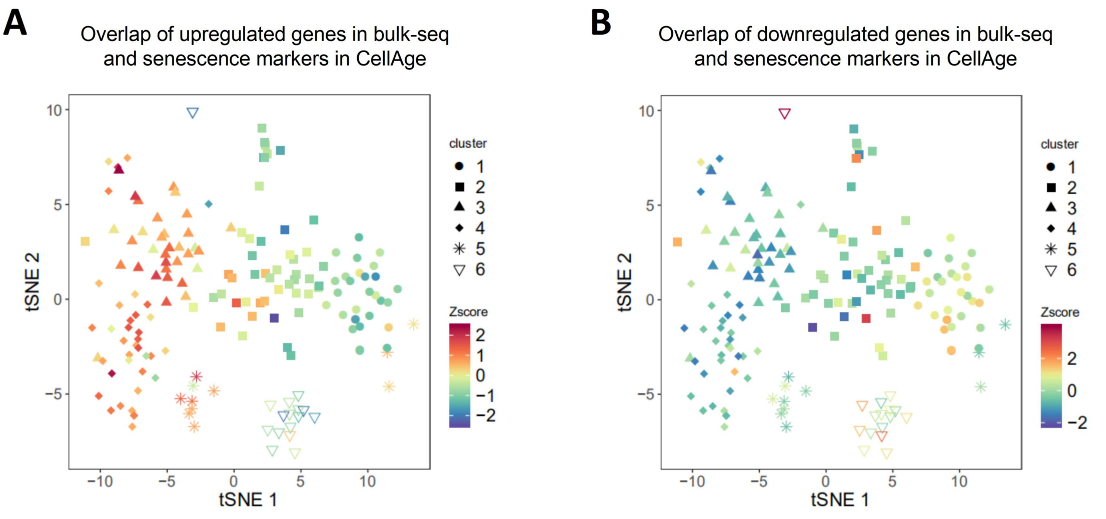

Fig s8. Continuously upregulated (A) and downregulated (B) genes in our previous PD6 to PD11 MEF bulk population RNA-seq data were overlapped with senescence-associated genes from CellAge database. And then the overall expression level of these overlapped genes in each single cell was presented in tSNE plot (Figure 1C). Z-score: Value calculated by Z normalization using AUC value (area under the curve); Bulk-seq: PD6 to PD11 MEF bulk population RNA-seq.

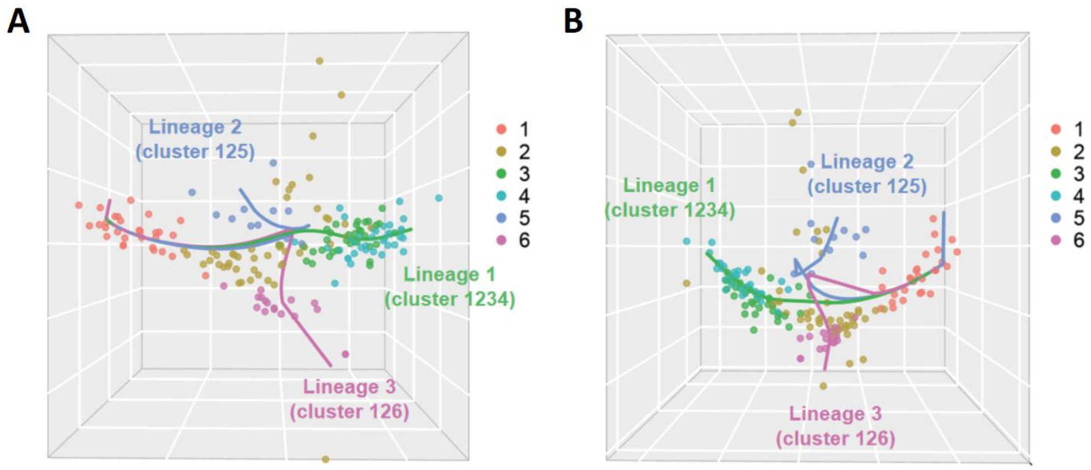

Fig s9. Trajectory analysis of single MEF transcriptomes using 5000 (A) or 8000 (B) highly variable genes. Three lineages obtained from analysis using 2556 genes were also supported by analysis using either 5000 or 8000 genes.

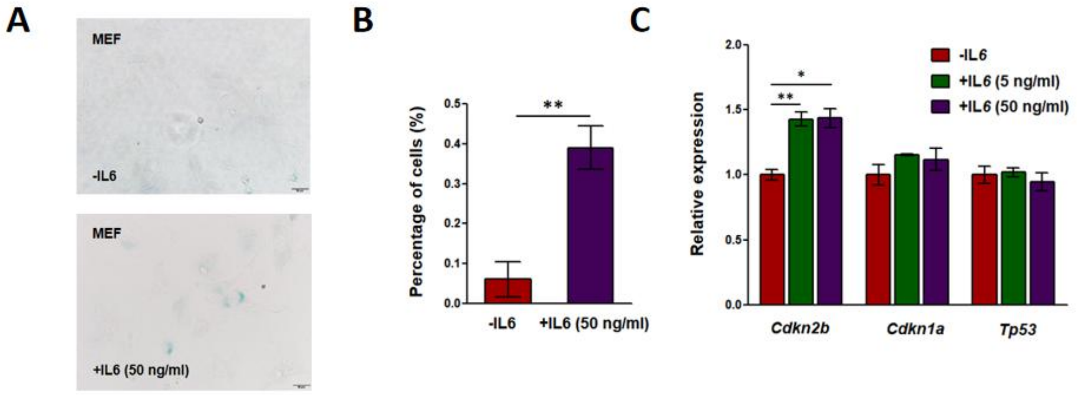

Fig s10. MEFs displayed partial senescence traits in medium containing IL6. (A,B) Deeper SA-β-Gal staining were observed in MEFs cultivated in medium with 50 ng/ml IL6 (A). Quantitative evaluation shown in panel B. (C) Moderate upregulation of *Cdkn2b* in MEFs cultivated in medium with 5 ng/ml or 50 ng/ml IL6. -IL6: MEFs cultivated in medium without IL6; +IL6(5ng)/+IL6(50ng): MEFs cultivated in medium with 5 ng/ml or 50 ngml IL6 respectively. * and ** represent p values less than 0.05 and 0.01 by *t*-test respectively.

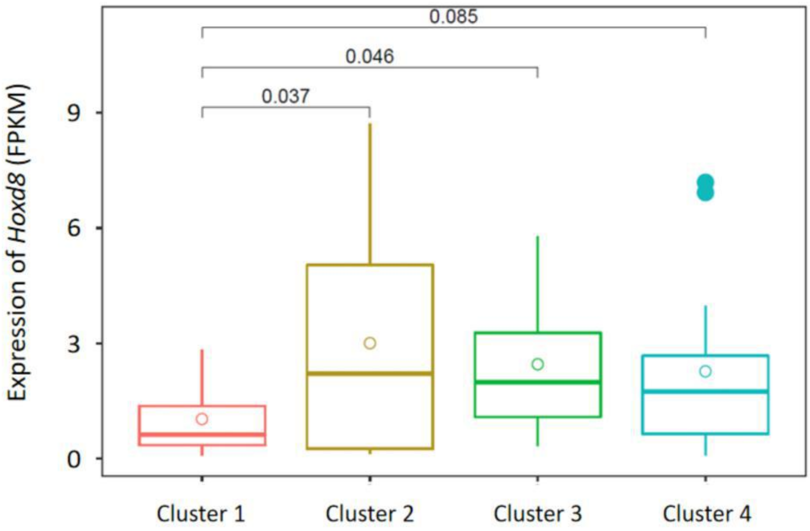

Fig s11. The overall expression level of *Hoxd8* was increased along lineage 1. Extreme value (FPKM > 10 or no expression detected) were filtered out. P-value of *Hoxd8* expression level between two clusters were displayed in figure. FPKM: Fragments Per Kilobase Million.

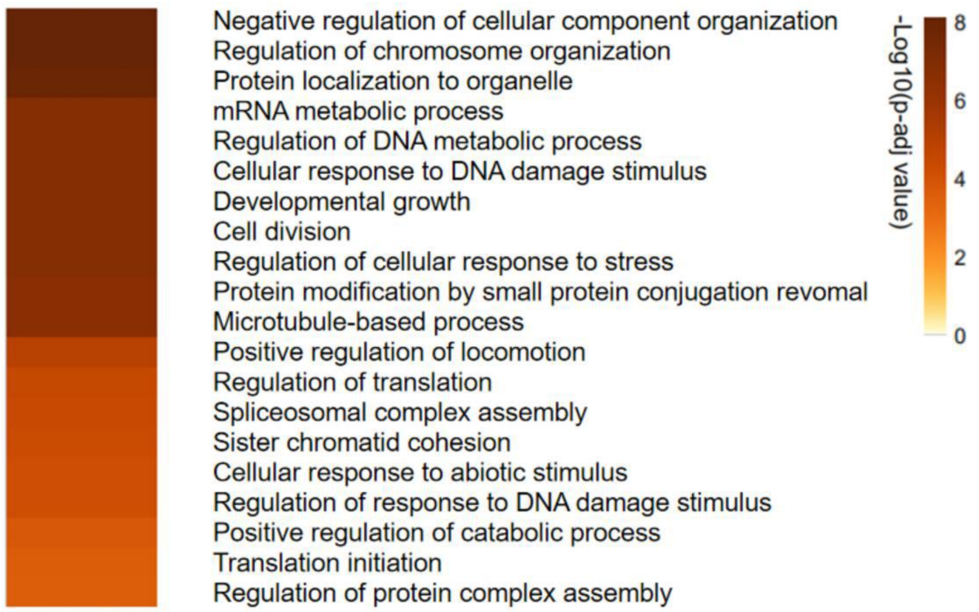

Fig s12. Heatmap showing top 20 GO terms enriched in Metascape using DEGs between *Hoxd8*-OE RNA-seq data and control NIH3T3 cells.

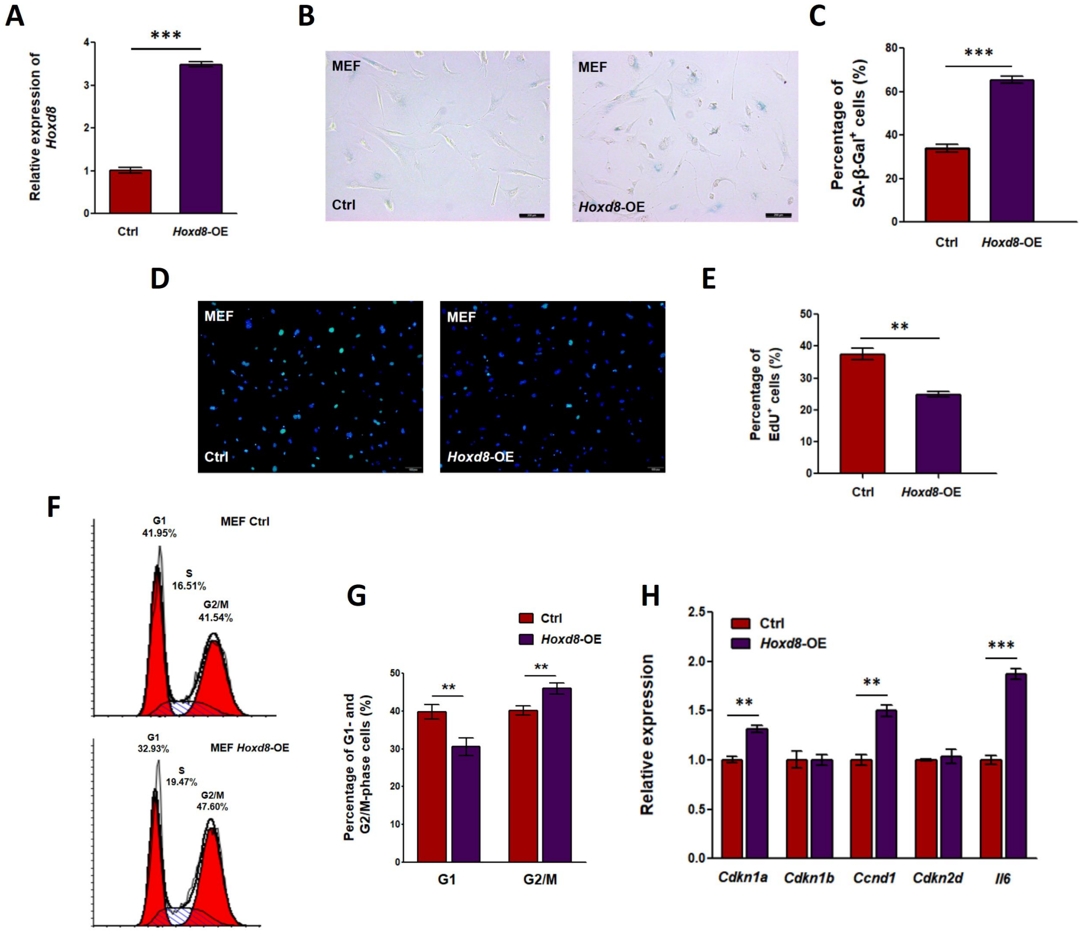

Fig s13. *Hoxd8*-OE MEFs exhibited a tendency of senescence. (A) OE of *Hoxd8* in MEFs was confirmed by qRT-PCR. *Gapdh* was used as internal control. (B, C) SA-β-Gal staining (B) and its quantitative evaluation (C) were compared between *Hoxd8*-OE and control MEFs. (D, E) EdU incorporation assay (D) and its quantitative evaluation (E) were compared between *Hoxd8*-OE and control MEFs. Blue: DAPI staining; Green: EdU incorporation. (F, G) Cell cycle analysis between *Hoxd8*-OE and control MEFs. F shows representative cell cycle distribution and G shows the quantitative evaluation. Different from NIH3T3 cells, OE of *Hoxd8* presumably triggered a G2 phase arrest in MEFs. (H) qRT-PCR analysis for cell cycle and senescence related genes in both *Hoxd8*-OE and control MEFs. Moderate upregulations of *Cdkn1a*, *Ccnd1*, and *Il6* were observed in *Hoxd8*-OE MEFs. *Gapdh* was used as internal control. *, **, and *** represent p values less than 0.05, 0.01 and 0.001 by t-test respectively.
